# The Functional Effects of Key Driver KRAS Mutations on Gene Expression in Lung Cancer

**DOI:** 10.3389/fgene.2020.00017

**Published:** 2020-02-04

**Authors:** Jisong Zhang, Huihui Hu, Shan Xu, Hanliang Jiang, Jihong Zhu, E. Qin, Zhengfu He, Enguo Chen

**Affiliations:** ^1^ Department of Pulmonary and Critical Care Medicine, Sir Run Run Shaw Hospital of Zhejiang University, Hangzhou, China; ^2^ Department of Anesthesiology, Sir Run Run Shaw Hospital of Zhejiang University, Hangzhou, China; ^3^ Department of Respiratory Medicine, Shaoxing People’s Hospital (Shaoxing Hospital, Zhejiang University School of Medicine), Shaoxing, China; ^4^ Department of Thoracic Surgery, Sir Run Run Shaw Hospital of Zhejiang University, Hangzhou, China

**Keywords:** Kirsten rat sarcoma oncogene (KRAS), mutation, lung cancer, predictor, gene expression

## Abstract

Lung cancer is a common malignant cancer. Kirsten rat sarcoma oncogene (KRAS) mutations have been considered as a key driver for lung cancers. KRAS p.G12C mutations were most predominant in NSCLC which was comprised about 11–16% of lung adenocarcinomas (p.G12C accounts for 45–50% of mutant KRAS). But it is still not clear how the KRAS mutation triggers lung cancers. To study the molecular mechanisms of KRAS mutation in lung cancer. We analyzed the gene expression profiles of 156 KRAS mutation samples and other negative samples with two stage feature selection approach: (1) minimal Redundancy Maximal Relevance (mRMR) and (2) Incremental Feature Selection (IFS). At last, 41 predictive genes for KRAS mutation were identified and a KRAS mutation predictor was constructed. Its leave one out cross validation MCC was 0.879. Our results were helpful for understanding the roles of KRAS mutation in lung cancer.

## Introduction

Lung cancer, known as a malignant cancer which defined as the overgrowth of uncontrolled cell in lung tissues, has proved be a key cause of cancer death. Each year, 1.3 million people die of lung cancer (Jemal et al., 2006; Jemal et al., 2011). Non-small-cell lung cancer (NSCLC) accounts for more than 85% of diagnosed lung cancer patients (Morgensztern et al., 2010). NSCLC can be further divided into adenocarcinoma, squamous cell carcinoma (SCC), and large cell carcinoma (Sandler et al., 2006; Morgensztern et al., 2010).

At present, the pathogenesis of lung cancer is not very clear, but is generally believed that one of the most important reason is the accumulation of mutations including single nucleotide transformation, small fragments of insertions and deletions, the changes of copy number, and chromosome rearrangement. Moreover, these mutations are closed with cell proliferation, invasion, metastasis, and apoptosis (Scagliotti et al., 2008; Liu et al., 2012). So, studying mutations in living systems will be helpful to understand how mutations are associated with lung-cancer biological processes.

In the last decade, researchers have uncovered the source of one of the important mutations is called as Kirsten rat sarcoma oncogene (KRAS) mutations in lung cancers using molecular studies (Gautschi et al., 2007). KRAS is the principal isoform of RAS. KRAS p.G12C mutations were most predominant in NSCLC which was comprised about 11–16% of lung adenocarcinomas (p.G12C accounts for 45–50% of mutant KRAS) (Cox et al., 2014). Other common KRAS mutations in lung cancer are G12V and G12D. In other cancers, such as pancreatic cancer and colorectal cancer, KRAS mutations are also frequent. Based on the TCGA data in cBioPortal (Gao et al., 2013), the most frequent KRAS mutations in pancreatic cancer are G12D, G12V, and G12R; the most frequent KRAS mutations in colorectal cancer are G12D, G12V, and G13D. KRAS may be a good lung cancer therapeutic target for searching potential drugs.

As above mentioned, mutations in KRAS is the most usual mutations that occur in lung cancer, especially in NSCLC (Mao et al., 1994; Mills et al., 1995; Nakamoto et al., 2001). KRAS mutation is more frequent in Caucasians than in Asians. Moreover, smokers may have more KRAS mutations than nonsmokers (Westcott and To, 2013; Ferrer et al., 2018). Single amino acid substitutions in codon 12 were most common KRAS mutations in NSCLC (Graziano et al., 1999). Therefore, the search for how the KRAS mutations affected the gene in lung cancer has been a long-standing goal in cancer biology.

In this study, to study the functional effects of key driver KRAS mutations on gene expression in lung cancer, we analyzed the gene expression profiles of 156 lung cancer cell lines with KRAS mutations and other 3,582 lung cancer cell lines without KRAS mutations. Forty-one discriminative genes for KRAS mutations were identified using two stage feature selection approach: (1) minimal Redundancy Maximal Relevance (mRMR) and (2) Incremental Feature Selection (IFS).

## Methods

### The Gene Expression Profiles of Cell Lines With and Without KRAS Mutations

To identify the key genes that distinguishes key driver KRAS mutations from other mutations, we downloaded the gene expression profiles of 156 lung cancer cell lines with KRAS mutations as positive samples and other 3,582 lung cancer cell lines without KRAS mutations as negative samples from publicly available Gene Expression Omnibus (GEO) database under accession number of GSE83744 (Berger et al., 2016). The expression levels of 978 representative genes from Broad Institute Human L1000 landmark were measured. The L1000 landmark was derived from the Connectivity Map (CMap) project (Subramanian et al., 2017). CMap is a large gene-expression dataset of human cells perturbed with many chemicals and genetic reagents (Lamb et al., 2006). These 1,000 genes were sensitive to perturbations and can reflect 81% of non-measured transcripts (Subramanian et al., 2017).

### Two Stage Feature Selection Approach

We applied two stage feature selection approach to select the biomarker genes. First, the genes were ranked based on not only their relevance with mutation samples, but also their redundancy among genes using the mRMR algorithm (Peng et al., 2005). It had a wide range of applications in bioinformatics for feature selection (Chen et al., 2018c; Chen et al., 2019e; Li and Huang, 2018; Li et al., 2019b; Wang and Huang, 2019a). As the equation shown below, Ω_*s*_, Ω_*t*_ and Ω were the set of m selected genes, n to-be-selected genes, and all m+n genes, respectively. We use mutual information (*I*) to measure the relevance of the expression levels of gene g from Ω*_t_* with KRAS mutation status t (Huang and Cai, 2013):/>

(1) D=I(g,t)

Meanwhile, the redundancy R of the gene g with the selected genes in Ω*_s_* can be calculated as below:

(2) $$\text{R}=\frac{1}{m}\left(\sum_{g_{i}\in {\text{Ω}}_{s}}I\left(g,g_{i}\right)\right)$$

The optimal gene *g_j_* from Ω*_t_* with max relevance with KRAS mutation status t and min redundancy with the selected genes in Ω*_s_* can be selected by maximizing mRMR function listed below

(3) $$\underset{g_{j}\in {\text{Ω}}_{t}}{\max}\left[I\left(g_{j},\text{t}\right)-\frac{1}{m}\left(\sum_{g_{i}\in {\text{Ω}}_{s}}I\left(g_{j},g_{i}\right)\right)\;\;\right]\;\left(j=1,2,\ldots ,n\right)$$

With N round evaluations, genes can be ranked as

(4) $$\text{S}=\left\{g_{1}^{'},g_{2}^{'},\ldots ,g_{h}^{'},\ldots ,g_{N}^{'},\right\}$$

The top ranked genes were associated with KRAS mutation status, and had little redundancy with other genes. Such genes were suitable for biomarkers. The top 200 genes were further analyzed at the second stage.

The second stage was to determine the number of selected genes using the IFS method (Chen et al., 2018b; Chen et al., 2019b; Chen et al., 2019c; Chen et al., 2019d; Chen et al., 2019f; Li et al., 2019a; Pan et al., 2019a; Pan et al., 2019b; ). To do so, 200 classifiers were constructed using top 1, top 2, top 200 genes. The LOOCV (leave-one-out cross validation) MCC (Mathew’s correlation coefficient) of the top k-gene classifier was calculated each time.

We tried several different classifiers: (1) SVM (Support Vector Machine) (Jiang et al., 2019; Yan et al., 2019; Chen et al., 2019a; Li et al., 2019a; Pan et al., 2019a; Wang and Huang, 2019b; Chen et al., 2019d), (2) 1NN (1 Nearest Neighbor) (Lei et al., 2013; Chen et al., 2016; Wang et al., 2017a), (3) 3NN (3 Nearest Neighbors), (4) 5NN (5 Nearest Neighbors), (5) Decision Tree (DT) (Huang et al., 2008; Huang et al., 2011; Chen et al., 2015), (6) Neural Network (NN) (Liu et al., 2017; Pan et al., 2018; Chen et al., 2019e). The function svm from R package e1071, function knn from R package class, function rpart from R package rpart, function nnet from R package nnet were used to apply these classification algorithms.

Based on the IFS curve in which x-axis was the number of genes and y-axis was the corresponding LOOCV MCC, we can decide the best gene combinations we should select. The peak of the curve was the optimal selection.

### Prediction Performance Evaluation of the Classifier

As we mentioned before, the prediction performance of each classifier was evaluated with leave-one-out cross validation (LOOCV) (Cui et al., 2013; Yang et al., 2014). It will go through N rounds and each sample will be tested during the N rounds. In each round, one sample will be tested using the model trained with the other N-1 samples. It can objectively evaluate all samples (Chou, 2011).

The performance metrics, including Sensitivity (Sn), Specificity (Sp), Accuracy (ACC), and Mathew’s correlation coefficient (MCC) were all calculated:

(5) $$S_{n}=\frac{TP}{TP+FN}$$

(6) $$S_{p}=\frac{TN}{TN+FP}\;\;\;$$

(7) $$ACC=\frac{TP+TN}{TP+TN+FP+FN}\;\;$$

(8) $$MCC=\frac{TP\times TN-FP\times FN}{\sqrt{\left(TP+FP\right)\left(TP+FN\right)\left(TN+FP\right)\left(TN+FN\right)}}$$

where TP, TN, FP, and FN stand for the number of true positive samples, true negative samples, false positive samples, and false negative samples, respectively. Since the sizes of KRAS mutation + samples and KRAS mutation - samples were imbalance and MCC can trade-off sensitivity and specificity (Chen et al., 2018a; Li et al., 2018; Pan et al., 2018; Pan et al., 2019a; Pan et al., 2019b), MCC was used as the main performance metric.

## Results and Discussion

### The Genes That Showed Different Expression Pattern Between KRAS Mutations From Other Mutations Samples

The top 200 most informative genes for KRAS mutations were identified using the mRMR method which has been widely used in bioinformatics filed (Zhao et al., 2013; Zhang et al., 2016). The C/C++ version software written by Peng et al. (Peng et al., 2005; Best et al., 2017) (http://home.penglab.com/proj/mRMR/) was used to apply the mRMR algorithm. Unlike the traditional statistical test based univariate feature selection methods, mRMR considers the relevance between gene expression and KRAS mutation status, and the redundancy among genes.

### The Optimal Biomarkers Identified From the mRMR Gene List With IFS Methods

**Figure 1:**
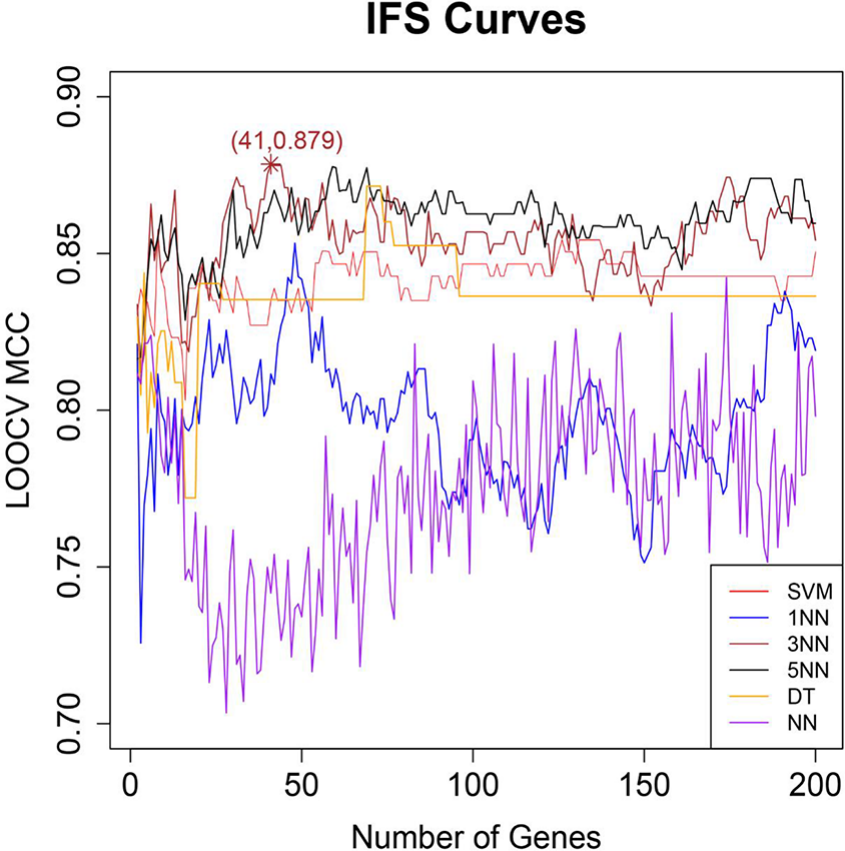
The IFS curves of six different classifiers. The x-axis was the number of genes and the y-axis was the then leave one out cross validation (LOOCV) MCC. The red, blue, brown, black, orange, and purple curves were the IFS results of SVM, 1NN, 3NN, 5NN, DT, and NN, respectively. Peak LOOCV MCCs of SVM, 1NN, 3NN, 5NN, DT, and NN were 0.858 with 8 genes, 0.853 with 48 genes, 0.879 with 41 genes, 0.878 with 59 genes, 0.871 with 69 genes, 0.842 with 174 genes. 3NN performed best. Therefore, the corresponding 41 genes were finally selected.

After genes were ranked by mRMR, the IFS procedure was applied to find the optimal number of genes to be selected. The IFS curve in **Figure 1** showed the relationship between the number of genes and their MCCs. The peak LOOCV MCCs of SVM, 1NN, 3NN, 5NN, DT, and NN were 0.858 with 8 genes, 0.853 with 48 genes, 0.879 with 41 genes, 0.878 with 59 genes, 0.871 with 69 genes, 0.842 with 174 genes. 3NN performed best. The corresponding 41 genes were shown in **Table 1**.

**Table 1:** The 41 genes selected by mRMR and IFS.

Rank	Gene	Rank	Gene
1	CTSL1	22	CCDC92
2	GNPDA1	23	BRP44
3	TRIB3	24	CDK19
4	STX1A	25	CD320
5	PHKA1	26	ATP1B1
6	CSNK1E	27	DRAP1
7	COL4A1	28	DUSP6
8	CEBPA	29	RAP1GAP
9	CEBPD	30	GALE
10	NSDHL	31	SSBP2
11	TP53	32	UBE2L6
12	MTHFD2	33	CCND3
13	RGS2	34	PAFAH1B1
14	NR3C1	35	RBM6
15	PPIC	36	C5
16	BAMBI	37	SDHB
17	PAK4	38	GRB10
18	FEZ2	39	UFM1
19	KTN1	40	ARL4C
20	HMGA2	41	PMAIP1
21	MMP1		

### The Prediction Metrics of the 41 Genes

The 41 genes were chosen with two stage feature selection methods: mRMR and IFS. To more carefully evaluate their prediction power, we checked their confusion matrix which showed the overlaps between actual KRAS mutation status and predicted KRAS mutation status using 3NN (**Table 2**). The LOOCV sensitivity, specificity, accuracy, and MCC were 0.840, 0.997, 0.991, and 0.879, respectively.

**Table 2:** The confusion matrix of actual sample classes and predicted sample classes using 3NN.

	Predicted KRAS mutation +	Predicted KRAS mutation −
Actual KRAS mutation +	131	25
Actual KRAS mutation −	10	3572
MCC = 0.879	Sensitivity = 0.840	Specificity = 0.997

### The Network Associations Between KRAS and the 41 Genes

**Figure 2:**
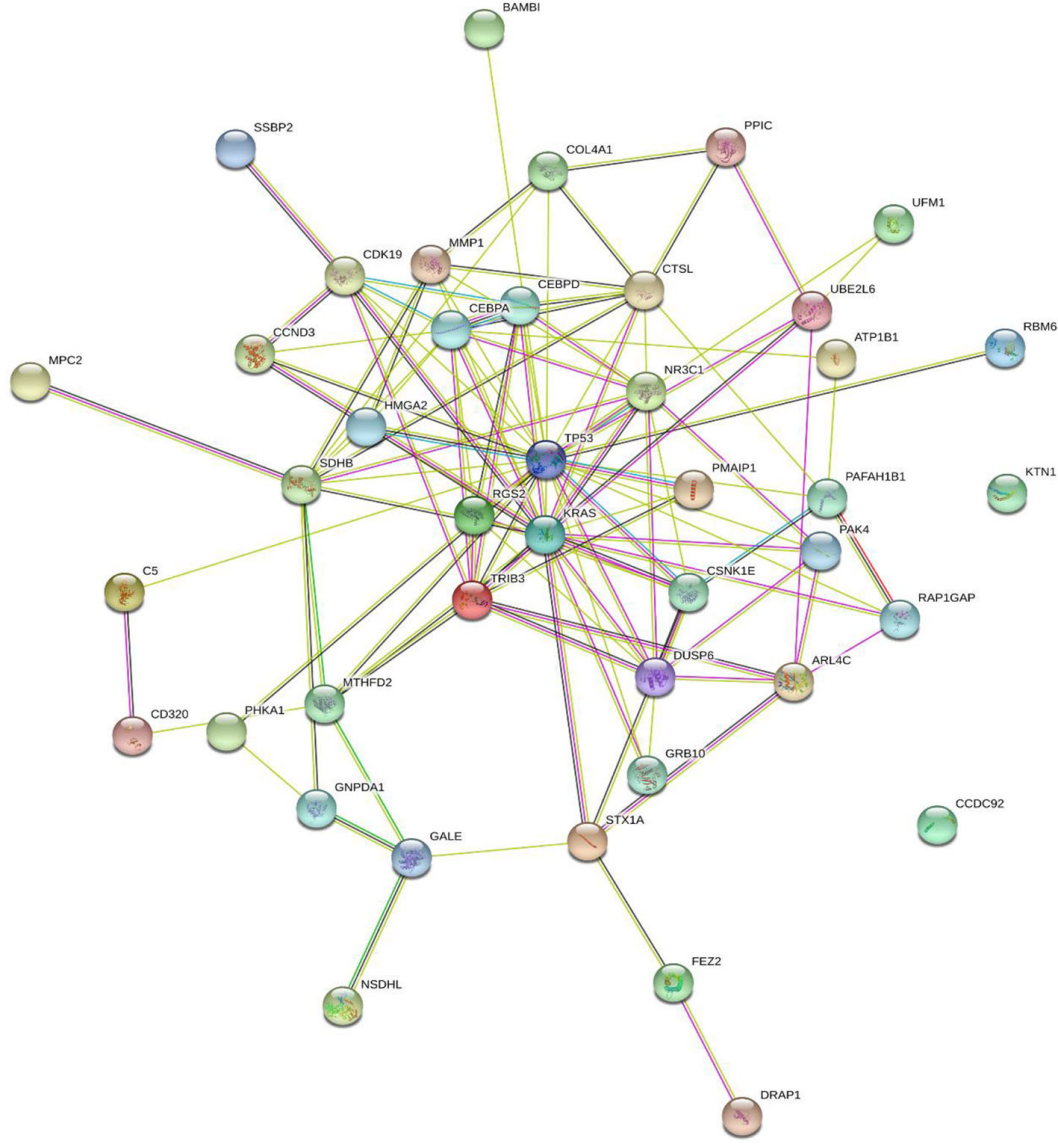
The functional association network of KRAS and the selected genes based on STRING database. Twenty out of 41 genes (CCND3, CDK19, CEBPA, CEBPD, CSNK1E, CTSL, DUSP6, GRB10, HMGA2, MMP1, MTHFD2, NR3C1, PAK4, PMAIP1, RAP1GAP, SDHB, STX1A, TP53, TRIB3, UBE2L6) had direct interactions with KRAS. Each line represented an interaction supported by different evidences. The skype-blue, purple, green, red, blue, grass green, black, and navy-blue edges were interactions from curated databases, experiment, gene neighborhood, gene fusions, gene co-occurrence, text mining, co-expression, and protein homology, respectively. For more detailed explanations, please refer to STRING database (https://string-db.org).

We searched KRAS and the eight genes in STRING database Version: 11.0 (https://string-db.org) and **Figure 2** showed their functional association networks. It can be seen that 20 out of 41 genes (CCND3, CDK19, CEBPA, CEBPD, CSNK1E, CTSL, DUSP6, GRB10, HMGA2, MMP1, MTHFD2, NR3C1, PAK4, PMAIP1, RAP1GAP, SDHB, STX1A, TP53, TRIB3, UBE2L6) had direct interactions with KRAS. The STRING network results supported that most of the 41 genes had direct interactions with KRAS.

### The Biological Significance of the Selected Genes in Lung Cancer

As mentioned earlier, we used mRMR algorithm and IFS program to screen out 41 genes which may be molecular markers for identifying KARS mutations. Subsequently, we reviewed studies of these genes in lung cancer and other cancers with high frequency of KARS mutations such as colorectal and pancreatic cancer. In the study of Zhang X et al., Tribbles-3 (TRIB3) pseudokinase can activate the β-catenin signal pathway, which in turn promotes the proliferation and migration of NSCLC cells (Zhang et al., 2019). In addition, blocking the activity of TRIB3 may be one of the mechanisms for the treatment of lung cancer (Ding et al., 2018). Wang X et al. have found that PAK4 is significantly associated with poor prognosis of NSCLC (Wang et al., 2016b), and LIMK1 phosphorylation mediated by it regulates the migration and invasion of NSCLC. Therefore, PAK4 may be an important prognostic indicator and a potential molecular target for treatment of NSCLC (Cai et al., 2015). HMGA2 affects apoptosis and is highly expressed in metastatic LUAD through Caspase 3/9 and Bcl-2. It is also considered to be a biomarker and potential therapeutic target for lung cancer therapy (Kumar et al., 2014; Gao et al., 2017b). A meta-analysis of lung cancer showed that metallo-proteinase 1 (MMP1)-16071G/2G polymorphism was a risk factor for lung cancer in Asians (Li et al., 2015). In addition, DUSP6 rs2279574 gene polymorphism is thought to predict the survival time of NSCLC patients after chemotherapy (Wang et al., 2016a). Cyclin D3 gene (CCND3) is a key cell cycle gene of NSCLC, which can promote the growth of LUAD (Zhang et al., 2017). Casein kinase I epsilon (CSNK1E), a circadian rhythm gene, whose genetic variation has a very significant correlation with the risk of lung cancer (Ortega and Mas-Oliva, 1986). CEPBA, can be used as a new tumor suppressor factor, Lu H et al. through clinical experiments, it was found that up-regulation of CEBPA is an effective method for the treatment of human NSCLC (Halmos et al., 2002; Lu et al., 2015). In addition, a comprehensive analysis of lung cancer genes by, Lv M shows that CEPBD may be involved in the development of lung cancer (Lv and Wang, 2015). TP53 mutation is very common in NSCLC and is considered to be a marker of poor prognosis and a prognostic indicator of lung cancer (Gao et al., 2017a; Labbe et al., 2017). Methylenetetrahydrofolate dehydrogenase 2 (MTHFD2) has redox homeostasis and can be used in the treatment of lung cancer (Nishimura et al., 2019). NR3C1 is reported to be involved in the pathways related to the biological process of lung cancer, and as a gene marker has a significant correlation with the survival of LUAD (Zhao et al., 2015; Luo et al., 2018). Cathepsin L1, as a protein was encoded by the CTSL1 gene, could reduce the cellular matrix and proteolytic cascades which resulting to promote invasion or metastatic activity (Duffy, 1996; Turk et al., 2012). Elevated expression of extracellular Cathepsin L was related with cancer progression of lung cancer cells (Okudela et al., 2016). Moreover, Cathepsin L is viewed as a downstream target of oncogenic KRAS mutations.

The above genes have not only been proved to be closely related to the prognosis, diagnosis, and treatment of lung cancer, but also have a direct interaction with KRAS. Some of the 41 selected genes have no direct interaction with KRAS, but are considered to be involved in the occurrence and development of lung cancer. RBM6 protein is located at 3p21.3, and its expression changes regulate many of the most common abnormal splicing events in lung cancer (Sutherland et al., 2010; Coomer et al., 2019). The double up-regulation of RGS2 gene is related to the poor overall survival rate of patients with lung adenocarcinoma (Yin et al., 2016). Epigenetic silencing of BAMBI has been identified as a marker of NSCLC, and overexpression of BAMBI may become a new target for the treatment of this cancer (Marwitz et al., 2016; Wang et al., 2017b). Overexpression of PAFA-H1B1 can lead to the occurrence and poor prognosis of lung cancer (Lo et al., 2012). Collagen alpha-1(IV) chain (COL4A1), encoded by the COL4A1 gene, was found previously to play a crucial role in the coordinating alveolar morphogenesis and formatting the epithelium vasculature lung tissue (Abe et al., 2017).

### The Potential Roles of the Selected Genes in Other Cancers

KRAS related genes are likely to be diagnostic, prognostic markers and therapeutic targets of lung cancer. We also looked for studies of these genes and KRAS high-frequency mutations in other cancers, mainly in colorectal and pancreatic cancer. According to Hua F et al., TRIB 3 gene knockout can reduce the occurrence of colon tumors in mice, reduce the migration of colorectal cancer cells, and reduce their growth in mouse transplanted tumors. The strategy of blocking the activity of TRIB3 can be used to treat colorectal cancer (Hua et al., 2019). Tyagi N et al. have found that PAK4 can maintain the stem cell phenotype of pancreatic cancer cells by activating STAT3 signal, which can be used as a new therapeutic target (Tyagi et al., 2016). TP53 mutation is associated with early stage of colorectal cancer (Laurent et al., 2011). There was a significant correlation between MMP1 and colon cancer mortality (Slattery and Lundgreen, 2014).

## Data Availability Statement

We downloaded the blood gene expression profiles of 156 KRAS mutations as positive samples and other 3582 mutations as negative samples from publicly available GEO (Gene Expression Omnibus) under accession number of GSE83744.

## Author Contributions

JZha conceived and designed the study. HH and SX performed data analysis. HJ wrote the paper. JZhu, EC and ZH reviewed and edited the manuscript. JZha approved final version of the manuscript. All authors read and approved the manuscript.

## Funding

This study was supported by the Funds from Science Technology Department of Zhejiang Province (LGF19H010010), Medical and Health Research Foundation of Zhejiang Province (2016ZDB005, 2017ZD020), China, WU JIEPING MEDICAL foundation (320.6750.19092-12), Beijing Xisike Clinical Oncology Research Foundation (Y-HS2017-037) and Medical Health and Scientific Technology Project of Zhejiang Province (2019RC182).

## Conflict of Interest

The authors declare that the research was conducted in the absence of any commercial or financial relationships that could be construed as a potential conflict of interest.
